# Treatment of Chronic Nasal Catarrh and Bronchitis

**Published:** 1883-05

**Authors:** J. G. Westmoreland

**Affiliations:** Atlanta, Ga.


### TREATMENT OF CHRONIC NASAL CATARRH AND BRONCHITIS.

By J. G. WESTMORELAND, M.D., Atlanta, Ga.

Like other chronic diseases, bronchitis, consumption and catarrh require the application of remedies to the diseased part. It is only by inhalation that the bronchia and lungs can be reached, and this is perhaps the best mode of impressing the nasal mucous membrane also But few remedies can be used in this way in sufficient strength to effect a cure, and from many years experience in treating various means, only one article has proved at all satisfactory. Very simple and cheap apparatus answers the purpose of carrying the remedy to the diseased part and arrestg the chronic inflammation. The treatment of bronchitis is often pursued for months with no other effect than the display of fine paraphernalia, while the suffering patient receives no essential benefit. Relief can be afforded by inhalation and the sufferer should have it in reality—not in form alone. I am a living monument of its salutary effects.

Any local alterative or catheretic will change the condition of a part, the subject of chronic inflammation, and when the diseased surface is external, little difficulty is had in the treatment. Very different is it, however, when the minute bronchia and lungs are to be impressed. Nitrate of silver, sulphate of copper, etc. may be made in the form of fine powder and inhaled, but they do not pass lower than the larynx, because when coming in contact with the moist surface they pass no further. Vapors of various substances have been extensively used without accomplishing the object. It is likely to condense in the mouth or throat, and if we should be fortunate enough to send it to the diseased part, it is in such diluted state that no good results.

Fumes make the only form in which a remedy can reasonably be expected to reach the small bronchia and lungs, and there is but one article belonging strictly to the class above alluded to, which is subject to this form. Rosin, sulphur, etc., when heated pass off in fumes, but they are not proper local alteratives for the purpose; iodine is an active remedy and passes into fumes readily when heated. A simple but altogether sufficient inhaler may be made of a small, wide-mouthed vial, such as is used for holding morphine. When heated, with a few grains of iodine in it, the fumes quickly appear and may be readily inhaled. The inhalation should be made through the nose, if disease exists in the nasal mucous membrane, otherwise the mouth will answer. The inhalation should be repeated in one to three days according to the amount of irritation caused by the remedy.

According to the length of time which chronic disease has existed, will it be more or less difficult to cure and more likely and repeatedly to return. It must be borne in mind, therefore, that the remedy must be reapplied as symptoms again appear.

